# La Telemedicina como herramienta para enfrentar la atención de pacientes durante el contexto de la COVID-19

**DOI:** 10.1016/j.aprim.2021.102061

**Published:** 2021-03-27

**Authors:** Luz Alexandra Javier Silva, Emilio Augusto Rosario Pacahuala

**Affiliations:** *Universidad Privada del Norte*, *Lima*, *Perú*

*Sr. Editor:*

La COVID-19 ha transformado la vida de las personas y el sector salud no está ajeno a ello, al contrario, el impacto en el personal médico, la infraestructura e incluso los protocolos de seguridad han tenido que ser replanteados, sin embargo, no se ha enfocado el interés sobre los pacientes.

Los usuarios del servicio de salud en muchos casos han decidido abandonar sus tratamientos por temor a contagiarse del SARS-CoV-2^1^, lo que ocasiona que los tratamientos preventivos, regenerativos entre otros, se hayan visto interrumpidos y han perjudicado la salud de muchos pacientes. Una de las medidas para evitar ello es la telemedicina, la cual fue puesta en práctica desde hace una década.

En estos últimos años ha ido incrementado su cobertura hasta convertirse en una alternativa para evitar la probabilidad de contagio de médicos y pacientes^2^. La aplicación de la medicina a distancia incluye las actividades como diagnóstico, tratamiento y prevención, formación médica continuada, investigación y evaluación^3^. Además de que cumple con el objetivo de mejorar la calidad del servicio porque facilita el acceso a la atención, reducción del tiempo y los costos de los servicios, y conserva la calidad diagnóstica^4^.

Los críticos manifiestan que las atenciones médicas pueden ocasionar un diagnóstico errado, debido a que se necesita un contacto con el paciente. No podemos negar que el servicio de telemedicina solo puede atender problemas básicos de salud o seguimiento de tratamiento, pero no podrá (por el momento) establecer un vínculo entre el paciente-profesional de salud. Asimismo es importante señalar que no basta con el conocimiento del especialista, además se necesita el fomento de las habilidades blandas para fortalecer estos vínculos.

Otra de las importantes ventajas es que permiten que las personas reciban un diagnóstico de un profesional de salud desde el lugar donde se encuentre. La falta de médicos en una determinada región puede ser cubierto por este tipo de servicio.

En conclusión, la telemedicina juega un rol importante frente al contexto, en especial para la población identificada como vulnerable. También se debe asumir como política de estado su implementación y redistribución económica para los pagos de los servicios^5^.
